# The writing on the arterial wall: epigenetic control of blood pressure and vascular remodeling

**DOI:** 10.1172/JCI194839

**Published:** 2025-06-02

**Authors:** Lloyd D. Harvey, Stephen Y. Chan

**Affiliations:** 1 Department of Medicine, and; 2 Specialty Training and Advanced Research (STAR) Program, David Geffen School of Medicine, UCLA, Los Angeles, California, USA.; 3 Center for Pulmonary Vascular Biology and Medicine, Pittsburgh Heart, Lung, and Blood Vascular Medicine Institute, Division of Cardiology, Department of Medicine, University of Pittsburgh School of Medicine and UPMC, Pittsburgh, Pennsylvania, USA.; 4 Division of Cardiology, University of Pittsburgh School of Medicine, Pittsburgh, Pennsylvania, USA.

## Abstract

Hypertension is a leading cause of morbidity and mortality, with pathologic consequences on multiple end-organ systems. Smooth muscle plasticity and its epigenetic regulation promote disease pathogenesis, but the genetic levers that control such activity are incompletely defined. In this issue of the *JCI*, Mangum et al. utilized high-density genomic data to define the causal and pathogenic role of a variant at the *JMJD3* locus — one that is associated with systolic blood pressure and governs an allele-specific molecular mechanism controlling smooth muscle behavior in hypertension. These findings have clinical implications relevant to patient risk stratification and personalized therapeutics.

## Smooth muscle cell plasticity is central to hypertension

**Figure 1:**
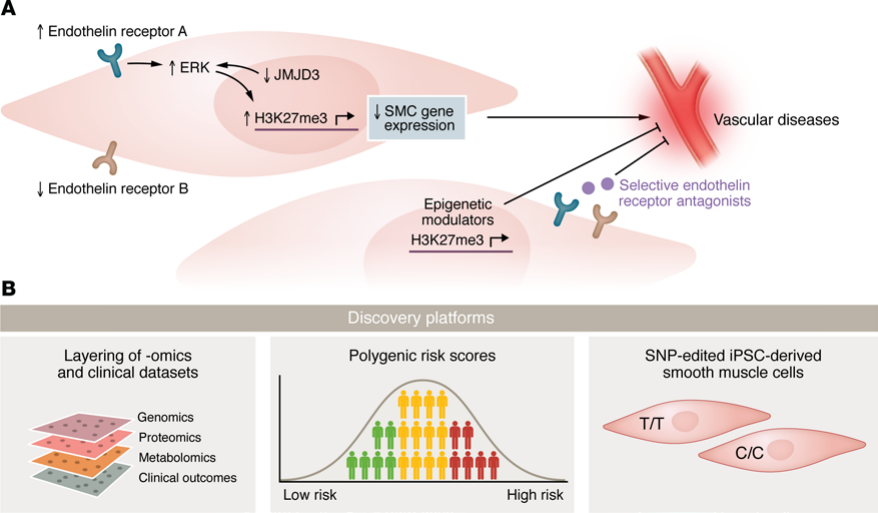
The single nucleotide variant in *JMJD3* links SMC pathobiology to vascular disease. (**A**) Activation of endothelin receptor A results in increased ERK signaling that is further enhanced by the loss of JMJD3. ERK signaling promotes H3K27me3 expression to attenuate SMC gene profiles with resultant cellular dysfunction. Selective endothelin receptor A antagonists or epigenetic modulators may serve as viable agents in reversing or preventing SMC dysfunction. (**B**) Integration of multiomics technologies can elucidate underlying mechanisms of disease with direct application to clinical outcomes. Further identification of genetic variants will have implications in polygenic risk scores in clinical prognostication. Identified variants can then be further studied at a molecular level, such as in inducible pluripotent stem cells (iPSCs) differentiated into SMCs for nuanced phenotyping. The implications could extend beyond hypertension to other disorders related to SMC dysfunction.

Uncontrolled hypertension is a substantial but potentially modifiable disease process and a primary cause of mortality worldwide (1). In the United States alone, hypertension contributes to more cardiovascular disease–related deaths than any other modifiable risk factor (2). Current antihypertensive agents can be effective, but are often inadequate, with nearly 100 million adults in the United States suffering from uncontrolled hypertension (2). Central to the pathogenesis of hypertension is smooth muscle cell (SMC) control of blood vessel contractility and relaxation, thus governing blood vessel tone (3). SMCs are highly plastic with the ability to switch between contractile (i.e., often associated with disease) and synthetic phenotypes in response to mechanical and molecular cues (4). SMC differentiation into the contractile phenotype is governed by transcription factors like serum response factor (SRF) and specificity protein 1 (SP1) (5, 6), whereas other transcription factors, such as Ets-1–like gene 1 (ELK1) and Krüppel-like factor 4 (KLF4), repress differentiation to generate the synthetic phenotype that contributes to diseases, including hypertension (7). The upstream mediators that activate these transcription factors are not well explored, especially in hypertension; however, there is an increasing suspicion for the role of epigenetic regulation and genetic variants in defining SMC phenotypes that ultimately may contribute to the pathogenesis of vascular disorders (8). In this issue of the *JCI*, Mangum et al. utilized population-level genomic data to identify a causal DNA variant associated with hypertension and further define the in vitro and in vivo epigenetic molecular mechanism dependent on said DNA variant in the pathogenesis of SMC dysfunction and ultimately hypertension (Figure 1) (9).

## JMJD3 governs SMC plasticity in hypertension

Analyzing multiple published GWAS, Mangum and colleagues concentrated on the previously identified locus of *JMJD3*, which encodes a histone demethylase with a known role in vascular disease (9–11). Statistical fine mapping of the *JMJD3* locus identified SNP rs62059712 as a high-confidence causal variant where the major T allele was associated with increased systolic blood pressure and located within a region displaying characteristics of an active transcription site. Using allelic variants of rs62059712 in luciferase reporter constructs transfected into human SMCs, the investigators demonstrated decreased transcriptional activity with the presence of the major T allele relative to the minor C allele. Notably, the region containing the C allele was predicted to bind the transcription factor SP1 that has a known role in SMC differentiation into the nonpathogenic contractile phenotype. This finding was subsequently confirmed with affinity purification of SP1 using DNA sequences containing the allelic variants and chromatin immunoprecipitation (ChIP).

To test the role for *JMJD3* in SMCs, the investigators generated *Jmjd3^fl/fl^Myh11^CreERT^* mice with implanted angiotensin II infusion pumps and measured blood pressure for two weeks. Mice with *Jmjd3*-deficient SMCs had higher blood pressures relative to both heterozygous and wild-type mice. To demonstrate that *JMJD3* controls SMC tone, the investigators applied GSJK4 — a JMJD3-specific inhibitor — to SMCs and observed a potent upregulation of activated myosin light chain 2 (pMLC2). RNA sequencing on isolated SMCs from *Jmjd3^fl/fl^Myh11^Cre+^* and *Jmjd3^fl/fl^Myh11^Cre–^* mice provided insights into the specific molecular mechanism. Mice with *Jmjd3*-deficient SMCs displayed downregulation of canonical SMC-specific genes, with concomitant upregulation of the SMC-repressing transcription factor *Klf4*. Furthermore, marked upregulation of the endothelin receptor A (*Ednra*) and downregulation of the endothelin receptor B (*Ednrb*) transcripts were observed. It is known that activated endothelin receptor A mediates potent SMC contraction, whereas endothelin receptor B activation is more complicated, with both contraction and relaxation effects (12). As such, the authors hypothesized that this difference in the endothelin receptor transcripts with *Jmjd3* deletion may underlie the hypertensive phenotype observed.

Mouse aortic SMCs transfected with *Jmjd3*-targeted RNAi demonstrated reduced *Ednrb*, whereas RNAi against *Sp1*, which is involved in normal SMC differentiation, resulted in increased *Ednra*. As expected, the RNAi against *Ednrb* upregulated genes involved in hypertension pathogenesis, and aortas isolated from *Jmjd3^fl/fl^Myh11^Cre+^* angiotensin II–treated mice had decreased *Ednrb* and increased *Ednra* expression. Overall, these studies suggest an intricate balance between the endothelin receptor subtypes mediated by JMJD3 (9).

Ex vivo, isolated SMCs from *Jmjd3^fl/fl^Myh11^Cre+^* mice were exposed to endothelin-1, angiotensin II, or phenylephrine. *Jmjd3*-deficient SMCs demonstrated increased vessel tone only with application of endothelin-1. The investigators further confirmed that endothelin-1–mediated vessel tone occurred through ERK activation — an established pathway in SMC contraction. Aortas isolated from *Jmjd3^fl/fl^Myh11^Cre+^* mice displayed increased vessel tone in response to endothelin-1, which was mitigated by the dual endothelin receptor antagonist bosentan. Correspondingly, administration of the endothelin receptor A–specific antagonist BQ-123 or the dual receptor antagonist in *Jmjd3^fl/fl^Myh11^Cre+^* mice showed normalization of blood pressures in comparison with wild-type controls (9).

To elucidate a mechanism by which JMJD3 controls hypertension, the authors observed an enrichment of the repressive epigenetic modulator H3K27me3 at the *Ednrb* promoter in *Jmjd3*-deficient SMCs. Additionally, *Jmjd3*-deficient SMCs showed increased H3K27me3 at other SMC-specific gene promoters, suggesting a failure of nonpathogenic SMC differentiation. A correlation between *JMJD3* and *EDNRB* expression was also seen in patients with hypertension by single-cell RNA sequencing. Thus, across multiple in vitro, in vivo, and ex vivo discovery platforms, the investigative team established that *JMJD3* positively regulates SMC identity, and *JMJD3* deficiency promotes dysfunctional SMCs and hypertension through H3K27me3 enrichment at SMC-specific promoters as well as through increased ERK activation (9).

## Implications and future directions

One of the most difficult translational challenges in the post-genomic era has been to ascribe a direct and causative pathobiological role to a given nucleotide variant(s) associated with a clinical metric or outcome. This challenge is further amplified in studies of non–protein-coding variants. In an experimental tour de force, Mangum et al. tackled this obstacle head-on in defining a complex genetic and epigenetic network of mechanisms to link a single nucleotide variant in *JMJD3* to SMC pathobiology and disease (9). At a fundamental level, this study offers an example of substantially advancing our understanding of genome-wide association with molecular mechanism, offering a roadmap of realizing genetic, molecular, and clinical integration. Their strategy is particularly applicable to the growing list of variants displaying genome-wide association with a host of cardiovascular conditions and human diseases. Yet, additional obstacles in this approach still exist, particularly for rare diseases, where limitations in the size of cohorts have prevented robust GWAS overlay and can require a more complex multiomics analysis to discern key mechanisms (13). Furthermore, heterogeneous diseases such as hypertension now are associated with thousands of genomic variants (14) and addressing how these variants may additively or synergistically control downstream hypertensive phenotypes would be crucial. However, it would be unreasonable to expect the level of experimental detail offered by Mangum and colleagues for each locus (9). The harmonization of datasets via more extensive open access and applications of artificial intelligence could aid further prioritization of key variants across this complicated landscape. Development of bioengineered discovery platforms that model the roles of patient-derived cells could also be important to study variants that are only found in humans but not rodent models.

More specifically for the *JMJD3* locus and rs62059712, this body of work could inform the development of more effective molecular diagnostics as well as individualized therapeutics (9). For example, the delineation of a functional mechanism for rs62059712 prioritizes SNP genotyping for diagnostic and prognostic purposes and could inform prioritization of loci in current polygenic risk scores for hypertension (15). Moreover, the link between *JMJD3* and endothelin biology offers biologic plausibility for the development of drugs in this space. Notably, the Federal Drug Administration recently approved the dual endothelin receptor antagonist aprocitentan for resistant hypertension, representing a drug class not previously developed for this condition. When coupled with the findings by Mangum et al., such endothelin-specific targeting could advance precision drugs — offering potentially more robust response in individuals harboring the T allele of rs62059712 where a genetically predisposed state drives higher levels of the endothelin receptor A. The investigators’ findings regarding the role of H3K27me3 in the regulation of *JMJD3* (9) could also prompt development of epigenetic drugs for hypertension, but challenges exist in off-target effects in this landscape. Finally, given the actions of *JMJD3* in SMC dysfunction (9), these findings could be relevant for other disorders dependent on this cell type, such as aneurysm formation, atherosclerosis, heart failure, reactive airway disease, and pulmonary arterial hypertension.

Consequently, despite the challenges ahead, the findings of Mangum and colleagues offer much needed guidance on effective strategies to define disease mechanisms of putative causal DNA variants, relevant to mosaic diseases like hypertension and beyond (9).

## Version 1 06/02/2025

Electronic publication
